# Endometrial cancer and patterns of use of oestrogen replacement therapy: a cohort study.

**DOI:** 10.1038/bjc.1989.91

**Published:** 1989-03

**Authors:** A. Paganini-Hill, R. K. Ross, B. E. Henderson

**Affiliations:** Department of Preventive Medicine, University of Southern California School of Medicine, Los Angeles 90033-0800.

## Abstract

5,160 non-hysterectomised women aged 44-100 years completed a health survey questionnaire as part of a longitudinal study of a southern California retirement community begun in June 1981. As of 1 January 1987, 50 incident cancers of the endometrium had occurred among these women, who had contributed 23,786 years of follow-up. Women who had used oestrogen replacement therapy had a relative risk of endometrial cancer of 10 compared to women who had never used oestrogens (P less than 0.0001). Risk increased with increasing duration of use (chi 2 test for trend = 50.60, P less than 0.0001); women who had used oestrogens for 15 or more years had a relative risk of 20 (95% C.I. = 7.2, 54) compared to non-users. While current and recent users (i.e. those who had used oestrogen within one year of the initial survey) had the greatest risk (RR = 25, 95% C.I. = 9.2, 69), women who had last used oestrogens 15 or more years ago still had a significantly increased risk (RR = 5.8, 95% C.I. = 2.0, 17). No other variable studied had a major effect on risk, except smoking. Women who smoked at the time of menopause had a significantly reduced risk of disease (RR = 0.38, P = 0.005), which was essentially unchanged after adjustment for oestrogen use.


*Br. J. Cancer* (1989), **59**, 445–447 © The Macmillan Press Ltd., 1989

### Endometrial cancer and patterns of use of oestrogen replacement therapy: a cohort study

A. Paganini-Hill, R.K. Ross & B.E. Henderson

*Department of Preventive Medicine, University of Southern California School of Medicine, 1441 Eastlake Avenue, Los Angeles, CA 90033-0800, USA.*

**Summary** 5,160 non-hysterectomised women aged 44–100 years completed a health survey questionnaire as part of a longitudinal study of a southern California retirement community begun in June 1981. As of 1 January 1987, 50 incident cancers of the endometrium had occurred among these women, who had contributed 23,786 years of follow-up. Women who had used oestrogen replacement therapy had a relative risk of endometrial cancer of 10 compared to women who had never used oestrogens ($P<0.0001$). Risk increased with increasing duration of use ($\chi^2$ test for trend = 50.60, $P<0.0001$); women who had used oestrogens for 15 or more years had a relative risk of 20 (95% C.I. = 7.2, 54) compared to non-users. While current and recent users (i.e. those who had used oestrogen within one year of the initial survey) had the greatest risk (RR = 25, 95% C.I. = 9.2, 69), women who had last used oestrogens 15 or more years ago still had a significantly increased risk (RR = 5.8, 95% C.I. = 2.0, 17). No other variable studied had a major effect on risk, except smoking. Women who smoked at the time of menopause had a significantly reduced risk of disease (RR = 0.38, $P=0.005$), which was essentially unchanged after adjustment for oestrogen use.

It is well established that post-menopausal women who use oestrogen replacement therapy are at increased risk of endometrial cancer (Smith *et al.*, 1975; Ziel & Finkle, 1975; Mack *et al.*, 1976; McDonald *et al.*, 1977; Gray *et al.*, 1977; Autunes *et al.*, 1979; Weiss *et al.*, 1979; Jick *et al.*, 1979; Jelovesk *et al.*, 1980; Shapiro *et al.*, 1980; Hulka *et al.*, 1980). Most studies which have explored this association have been of the case-control design. The appropriate control series for such studies has been the topic of considerable scientific concern (Horwitz & Feinstein, 1978). Few prospective studies have evaluated the strength of this association (Hoover *et al.*, 1976; Persson *et al.*, 1986) and few of the case-control studies have contributed information on risk after lengthy periods off therapy. This paper reports the results of a large cohort study in which the prevalence of past oestrogen use was high.

### Methods

In June 1981 a health questionnaire was mailed to all residents of Leisure World, Laguna Hills, a retirement community near Los Angeles, California. New residents who moved into the community after this date were mailed the questionnaire in June 1982, June 1983 and October 1985. Residents of this community are almost entirely white, moderately affluent and well educated. The residents' median age was 73 years at the time of the initial mailing and about two-thirds were women. After three mailings, 13,986 (61%) of the 22,781 residents returned questionnaires; 8,882 of these respondents were women.

The health questionnaire requested information on certain prior medical diagnoses including cancer; height and weight; use of cigarettes and alcohol; and for women, menstrual and reproductive events, including gynaecological surgeries. Detailed information was collected on use of oestrogens during menopause including routes of administration (injectable, oral, vaginal) and duration of therapy. For the most commonly used oral oestrogen, Premarin, pill colour was used to help identify the dosage(s) taken.

Pathological diagnosis of cancer among cohort members are obtained from five local hospitals. Surveys among community residents suggest that over 85% of inpatient hospital care occurs in these hospitals. The cohort is also followed for deaths using the death certificate records of the local county health department. Death certificates are obtained for additional decedents identified by the community business office, from the obituary columns of the local newspaper, and from information provided by relatives and friends. In addition, we have conducted a biennial remailing to the cohort. To date only 13 cohort members have been lost to follow-up; search of the National Death Index did not reveal that these individuals were deceased.

Age-adjusted mortality rates were computed by direct standardisation using an internal standard (i.e. the person-years distribution of the total cohort under study) and four age groups. Relative risks and $P$ values were obtained using a regression method that assumed that the occurrence of death could be regarded as a Poisson process with a constant hazard rate for a given person (Breslow *et al.*, 1980). The GLIM statistical software package program (Royal Statistical Society, 1978) was used to make these calculations. All reported $P$ values are two-sided.

### Results

As of 1 January 1987, 50 women had been diagnosed as having endometrial cancer among the 5,160 women who indicated they had not had uterine cancer and/or a hysterectomy on the initial questionnaire. Seven of these 5,160 women provided no information on oestrogen use and were excluded from further analyses related to this variable. The age-specific and age-adjusted rates for endometrial cancer by history of oestrogen use are given in Table I. There were 45 endometrial cancers among the women who had used oestrogen replacement therapy and five among those who had never used oestrogens, yielding an age-adjusted relative risk (RR) of 10 ($P<0.0001$).

Duration was evaluated as the total number of years of all types of oestrogen replacement therapy, regardless of route of administration. Dose was only available for women taking oral conjugated oestrogens. The reported dose is that taken for the longest period of time. Eighty-nine per cent of oestrogen users had used oral oestrogen for at least part of the time, and 62% had used only this form of oestrogen replacement therapy.

Years since last oestrogen use was strongly associated with risk of endometrial cancer ($\chi^2$ for trend = 51.82, $P<0.0001$). The highest risk was found in women who were currently using oestrogen or who had taken it within one year of completing the original questionnaire (RR = 25 compared to

Correspondence: A. Paganini-Hill.
Received 28 June 1988; and in revised form 2 November 1988.

**Table I** Age-adjusted endometrial cancer incidence rates per 1,000 and number of cases by use of oestrogen replacement therapy

| | Woman-years | Number of cases | Incidence rate | Relative risk |
|---|---|---|---|---|
| Any oestrogen use | | | | |
| Never | 12,472 | 5 | 0.3 | 1.0 |
| Ever | 11,281 | 45 | 2.9 | 10 |
| Duration of oestrogen use | | | | |
| ≤2 | 3,888 | 8 | 2.1 | 5.2 |
| 3–7 | 2,607 | 7 | 3.1 | 7.0 |
| 8–14 | 2,336 | 13 | 4.9 | 4 |
| 15+ | 2,134 | 17 | 7.6 | 20 |
| Years since cessation of oestrogen | | | | |
| 15+ | 4,202 | 10 | 2.1 | 5.8 |
| 8–14 | 2,372 | 7 | 2.7 | 8.1 |
| 2–7 | 2,366 | 10 | 3.4 | 12 |
| 0–1 | 2,076 | 18 | 8.4 | 25 |
| Dose of oestrogen | | | | |
| ≤0.625 | 3,423 | 20 | 5.3 | 15 |
| ≥1.25 | 2,996 | 13 | 4.1 | 11 |

never users, 95% C.I. = 9.2, 69). However, women who had stopped taking oestrogens as long ago as 15 years still had a RR of 5.8 (95% C.I. = 2.0, 17) compared to non-users.

Duration of oestrogen replacement therapy was also strongly related to risk of endometrial cancer. Risk increased significantly with increasing duration of use ($\chi^2$ for trend = 50.60, $P<0.0001$). Women who had taken oestrogen for 15 or more years had a risk of 20 compared to never users (95% C.I. = 7.2, 54).

Ninety-two per cent of the women who reported having taken Premarin were able to report the dosage(s) taken (78% of oral oestrogen users reported they had taken Premarin). Women who had used primarily a high pill dose (≥1.25 mg conjugated equine oestrogen) had a relative risk similar to that of women who had used a lower dose. However, we had only 13 endometrial cancer cases among the high dose group and 20 cases among the lower dose group. In both groups the highest risk was seen for long-term and recent users of oestrogen.

Data on intervals after cessation of oestrogen use, stratified according to duration of use, are presented in Table II. Both duration and time since last use were independently related to risk of endometrial cancer. The highest risk (RR = 34) was observed for recent long-term users (15+ years).

No other variable studied (age at menarche, parity, number of children, age at last menstrual period, weight, alcohol use) was significantly related to the development of endometrial cancer except smoking and years since last PAP test (Table III). Women who were smokers at the time of their last menstrual period had a RR of 0.38 compared to non-smokers ($P=0.006$). After adjusting for oestrogen use,

**Table II** Age-adjusted endometrial cancer incidence rates per 1,000 and number of cases by duration of and years since cessation of oestrogen replacement therapy

| Duration | Years since cessation | Woman-years | Number of cases | Incidence rate | Relative risk |
|---|---|---|---|---|---|
| None | No use | 12,472 | 5 | 0.3 | 1.0 |
| ≤3 | 0–1 | 352 | 0 | – | – |
| | 2–14 | 1,354 | 4 | 3.7 | 8.8 |
| | 15+ | 2,776 | 7 | 2.3 | 6.2 |
| 4–14 | 0–1 | 821 | 7 | 8.1 | 27 |
| | 2–14 | 2,313 | 8 | 2.6 | 9.8 |
| | 15+ | 1,189 | 2 | 1.3 | 4.0 |
| 15+ | 0–1 | 865 | 11 | 11.8 | 34 |
| | 2–14 | 1,056 | 5 | 4.8 | 12 |
| | 15+ | 213 | 1 | 2.5 | 7.2 |

**Table III** Age-adjusted endometrial cancer incidence rates per 1,000 and number of cases by other potential risk factors

| | Woman-years | Number of cases | Incidence rate | Relative risk | |
|---|---|---|---|---|---|
| Weight (lb) | | | | | |
| ≤120 | 7,928 | 17 | 2.1 | 1.00 | |
| 121–138 | 7,968 | 19 | 2.4 | 1.10 | |
| 139+ | 7,834 | 14 | 1.8 | 0.82 | |
| Weight at last menstrual period (lb) | | | | | |
| ≤122 | 7,857 | 15 | 1.9 | 1.00 | |
| 123–135 | 7,842 | 25 | 3.2 | 1.68 | |
| 136+ | 6,889 | 9 | 1.3 | 0.70 | |
| Years since last PAP test | | | | | |
| ≤1 | 14,712 | 39 | 2.6 | 1.00 | |
| 2–5 | 5,422 | 9 | 1.6 | 0.63 | |
| 6+ | 2,111 | 1 | 0.5 | 0.19 | $P=0.02$[a] |
| Smoking at last menstrual period | | | | | |
| No | 16,218 | 42 | 2.7 | 1.00 | |
| Yes | 7,475 | 8 | 0.9 | 0.38 | $P=0.005$ |

[a]Test for trend.

this RR was essentially unchanged (RR = 0.36) and statistically significant ($P=0.004$). Years since cessation of oestrogen use was highly correlated with years since last PAP test. Some 87% of women who had used oestrogen within the last year had had a PAP test within the last year versus 81% of women who had used ERT 2–7 years ago, 66% of women who had used ERT 8 or more years ago and 59% of women who had never used ERT.

The five non-oestrogen users who developed endometrial cancer were all over 80 years of age at the time of diagnosis and all were non-smokers at the time of menopause (versus 71% of the women without endometrial cancer who had never used oestrogen). Three cases had never been pregnant versus 28% of the non-cases. Otherwise there were no outstanding differences between cases and non-cases among the non-users of oestrogen.

Ten women diagnosed with endometrial cancer have died (three non-oestrogen users), six from their endometrial cancer (two non-oestrogen users), two from ischaemic heart disease (one non-oestrogen user), one from bronchiectasis and one from lymphoma. The 3-year survival rate was 85% and 60% for oestrogen users and non-users, respectively.

### Discussion

While our data on both oestrogen use and other variables are self-reported, we have evidence that these data are both reliable and valid (Paganini-Hill & Ross, 1982). Previous studies of oestrogen use in this community have found extremely good correlation in relative risks for various chronic diseases when oestrogen use was ascertained by interviews, medical records or pharmacy records (Mack *et al.*, 1976; Ross *et al.*, 1980; Paganini-Hill *et al.*, 1981).

This study confirms in a population-based cohort that the risk of endometrial cancer increases sharply with increasing duration of usage of oestrogen replacement therapy. We found no clear effect of pill dose on risk. Although this did not appear to be due to longer duration of use in women using the lower pill dose, the number of cases did not permit a detailed evaluation of this proposition. Twenty-six per cent of Premarin users in our cohort reported using multiple pill doses. The risk remains substantially elevated even after 15 years since cessation of use. Other reports have shown that the elevated risk persists after cessation of oestrogen use (Mack *et al.*, 1976; Weiss *et al.*, 1979; Shapiro *et al.*, 1985), but this study extends the risk to longer drug-free intervals. One previous study suggested that the risk of endometrial cancer abates rapidly within two years since cessation of use (Hulka *et al.*, 1980), but most oestrogen use in that study was of short duration (<3 years). Because of the longlasting effect of oestrogen use on the risk of endometrial cancer, it is imperative that women who have used oestrogen replacement therapy, especially for long periods of time, be continually followed gynaecologically. Our results pertain almost exclusively to oral oestrogens used as conjugated equine preparations. Combination hormone replacement therapy (oestrogen plus progestin) is a relatively recent alternative therapy in the USA. Only about 1% of women in our cohort have ever used such treatment.

The observation that cigarette smoking is protective against the development of endometrial cancer is not surprising. Several recent case-control studies have reported similar results (Baron *et al.*, 1986; Lesko *et al.*, 1985). This may be due to an anti-oestrogenic effect of smoking. Women who smoke have lower urinary excretion rates of endogenous oestrogens (MacMahon *et al.*, 1982; Jensen *et al.*, 1984) and lower serum concentrations of oestrogens during oestrogen therapy (Jensen *et al.*, 1984).

Surprisingly, we found no strong evidence that high weight is associated with an elevated risk of endometrial cancer in this population. Our ability to address adequately this association was limited by the small number of cases occurring in women who had never used oestrogen replacement therapy ($n = 5$). Cohort members tended to fall within a rather narrow weight range and few are in the upper extreme of weight.

Our finding of a reduced risk with increased time since last PAP test was also somewhat unexpected. Women in this community who have PAP tests regularly are more likely to use oestrogen than women who do not. After adjusting for oestrogen use, the relative risk estimates for years since last PAP test were attenuated and the trend was no longer statistically significant.

Our finding of better survival among oestrogen users relative to non-users is consistent with other studies (Underwood *et al.*, 1979; Robboy *et al.*, 1979; Elmwood & Boyes, 1980; Collins *et al.*, 1980; Chu *et al.*, 1982; Schwartzbaum *et al.*, 1987). The reason for this phenomenon is not entirely understood but may be due to an earlier diagnosis of endometrial cancer, on average, in oestrogen users because of more aggressive and complete medical surveillance. Our data provide some support for this hypothesis. Only eight of the 50 cases in our cohort were non-localised at diagnosis. As observed by others (Mack *et al.*, 1976; Jelovesk *et al.*, 1980), the association with oestrogen use for non-localised disease, while elevated, was substantially reduced (RR = 2.0) in comparison to that for localised cancer.

The authors gratefully acknowledge the invaluable assistance of their research staff, Mary Arthur, Ann Chao and Beverly Ducey, and are indebted to the residents of Leisure World, Laguna Hills, whose cooperation made this work possible. Supported by grants CA32197, CA00652 and CA17054 from the National Cancer Institute, National Institutes of Health, Bethesda, MD.

### References

ANTUNES, C.M.F., STOLLEY, P.D., ROSENSHEIN, N.B. *et al* (1979). Endometrial cancer and estrogen use: report of a large case-control study. *N. Engl. J. Med.*, **300**, 9.

BARON, J.A., BYERS, T., GREENBERG, E.R. *et al.* (1986). Cigarette smoking in women with cancers of the breast and reproductive organs. *JNCI*, **77**, 677.

BRESLOW, N.E., LUBIN, J.H., MAREK, P. & LANGHOLZ, B. (1980). Multiplicative models and cohort analysis. *J. Am. Stat. Assoc.*, **78**, 1.

CHU, J., SCHWEID, A.I. & WEISS, N.S. (1982). Survival among women with endometrial cancer: a comparison of oestrogen users and nonusers. *Am. J. Obstet. Gynecol.*, **143**, 569.

COLLINS, J., DONNER, A., ALLEN L.H. *et al.* (1980). Oestrogen use and survival in endometrial cancer. *Lancet*, **ii**, 96.

ELWOOD, J.M. & BOYES, D.A. (1980). Clinical and pathological features and survival of endometrial cancer patients in relation to prior use of estrogens. *Gynecol. Oncol.*, **10**, 173.

GRAY, L.A. SR, CHRISTOPHERSON, W.M. & HOOVER, R.N. (1977). Estrogens and endometrial carcinoma. *Obstet. Gynecol.*, **49**, 385.

HOOVER, R., FRAUMENI, J.F., EVERSON, R. *et al.* (1976). Cancer of the uterine corpus after hormonal treatment for breast cancer. *Lancet*, **i**, 885.

HORWITZ, R.I. & FEINSTEIN, A.R. (1978). Alternative analytic methods for case-control studies of estrogens and endometrial cancer. *N. Engl. J. Med.*, **299**, 1089.

HULKA, B.S., FOWLER, W.C., KAUFMAN, D.G. *et al.* (1980). Estrogen and endometrial cancer: cases and two control groups from North Carolina. *Am. J. Obstet. Gynecol.*, **137**, 92.

JELOVESK, F.R., HAMMOND, C.B., WOODARD, B.H. *et al.* (1980). Risk of exogenous estrogen therapy and endometrial cancer. *Am. J. Obstet. Gynecol.*, **137**, 85.

JENSEN, J., HUMMER, L., CHRISTIANSEN, C. *et al.* (1984). Cigarette smoking, serum estrogens, and bone loss in early postmenopausal women during hormone replacement therapy. In *Osteoporosis: Proceedings of the Copenhagen Symposium on Osteoporosis June 3–8, 1984*, Christiansen, C., Arnaud, C.D., Nordin, B.E.C. *et al.* (eds) p. 401. Department of Clinical Chemistry, Glostrup Hospital, Denmark.

JICK, H., WATKINS, R.N., HUNTER, J.R. *et al.* (1979). Replacement estrogens and endometrial cancer. *N. Engl. J. Med.*, **300**, 218.

LESKO, S.M., ROSENBERG, L., KAUFMAN, D.W. *et al.* (1985). Cigarette smoking and the risk of endometrial cancer. *N. Engl. J. Med.*, **313**, 593.

MACK, T.M., PIKE, M.C., HENDERSON, B.E. *et al.* (1976). Estrogens and endometrial cancer in a retirement community. *N. Engl. J. Med.*, **294**, 1262.

MACMAHON, B., TRICHOPOULOS, D., COLE, P. *et al.* (1982). Cigarette smoking and urinary estrogens. *N. Engl. J. Med.*, **307**, 1062.

MCDONALD, T.W., ANNEGER, J.F., O'FALLON, W.M. *et al.* (1977). Exogenous estrogen and endometrial carcinoma: case control and incidence study. *Am. J. Obstet. Gynecol.*, **127**, 572.

PAGANINI-HILL, A. & ROSS, R.K. (1982). Reliability of recall of drug usage and other health related information. *Am. J. Epidemiol.*, **116**, 114.

PAGANINI-HILL, A., ROSS, R.K., GERKINS, V.R. *et al.* (1981). A case-control study of menopausal estrogen therapy and hip fractures. *Ann. Intern. Med.*, **95**, 28.

PERSSON, I.R., ADAMI, H-O., EKLUND, G., JOHANSSON, E.D.B., LINDBERG, B.S. & LINDGREN, A. (1986). The risk of endometrial neoplasia and treatment with estrogens and estrogen-progestogen combinations. *Acta Obstet. Gynecol. Scand.*, **65**, 211.

ROBBOY, S.J. & BRADLEY, R. (1979). Changing trends and prognostic features in endometrial cancer associated with exogenous estrogen therapy. *Obstet. Gynecol.*, **54**, 269.

ROSS, R.K., PAGANINI-HILL, A., GERKINS, V.R. *et al.* (1980). A case-control study of menopausal estrogen therapy and breast cancer, *JAMA*, **243**, 1635.

ROYAL STATISTICAL SOCIETY (1978). Generalized Linear Interactive Modelling, The GLIM System, Release 3. Numerical Algorithms Group: Oxford.

SCHWARTZBAUM, J.A., HULKA, B.S., FOWLER, W.C. JR *et al.* (1987). The influence of exogenous estrogen use on survival after diagnosis of endometrial cancer. *Am. J. Epidemiol.*, **126**, 851.

SHAPIRO, M.B., KAUFMAN, M.S., SLONE, D. *et al.* (1980). Recent and past use of conjugated estrogens in relation to adenocarcinoma of the endometrium. *N. Engl. J. Med.*, **303**, 485.

SHAPIRO, S., KELLY, J.P., ROSENBERG, L. *et al.* (1985). Risk of localized and widespread endometrial cancer in relation to recent and discontinued use of conjugated estrogens. *N. Engl. J. Med.*, **313**, 969.

SMITH, D.C., PRENTICE, R., THOMSON, D.J. *et al.* (1975). Association of exogenous estrogen and endometrial cancer. *N. Engl. J. Med.*, **293**, 1164.

UNDERWOOD, P.B. JR, MILLER, C.M., KREUINER, A. JR *et al.* (1979). Endometrial carcinoma: the effects of estrogens. *Gynecol. Oncol.*, **8**, 69.

WEISS, N.S., SZEKELY, D.R., ENGLISH, D.R. *et al.* (1979). Endometrial cancer in relation to patterns of menopausal estrogen use. *JAMA*, **242**, 261.

ZIEL, H.K. & FINKLE, W.D. (1975). Increased risk of endometrial carcinoma among users of conjugated estrogens. *N. Engl. J. Med.*, **293**, 1167.


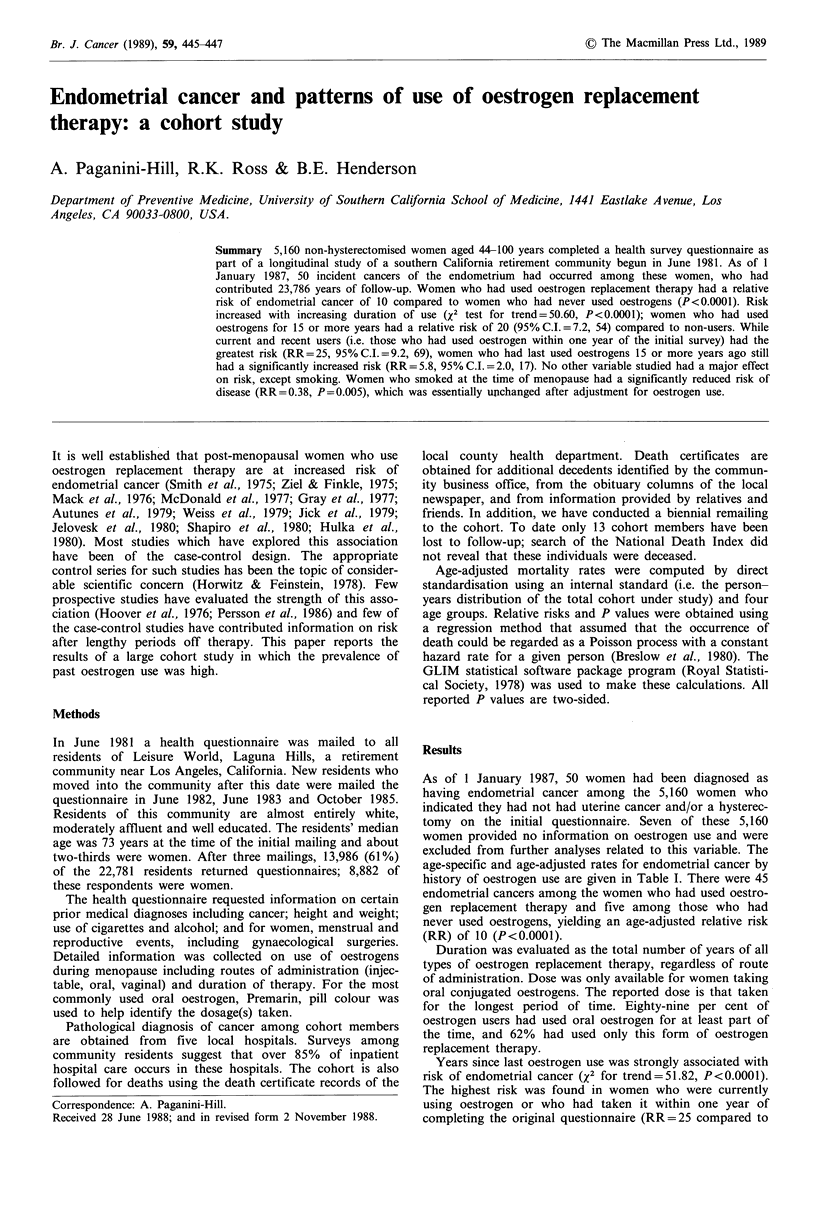

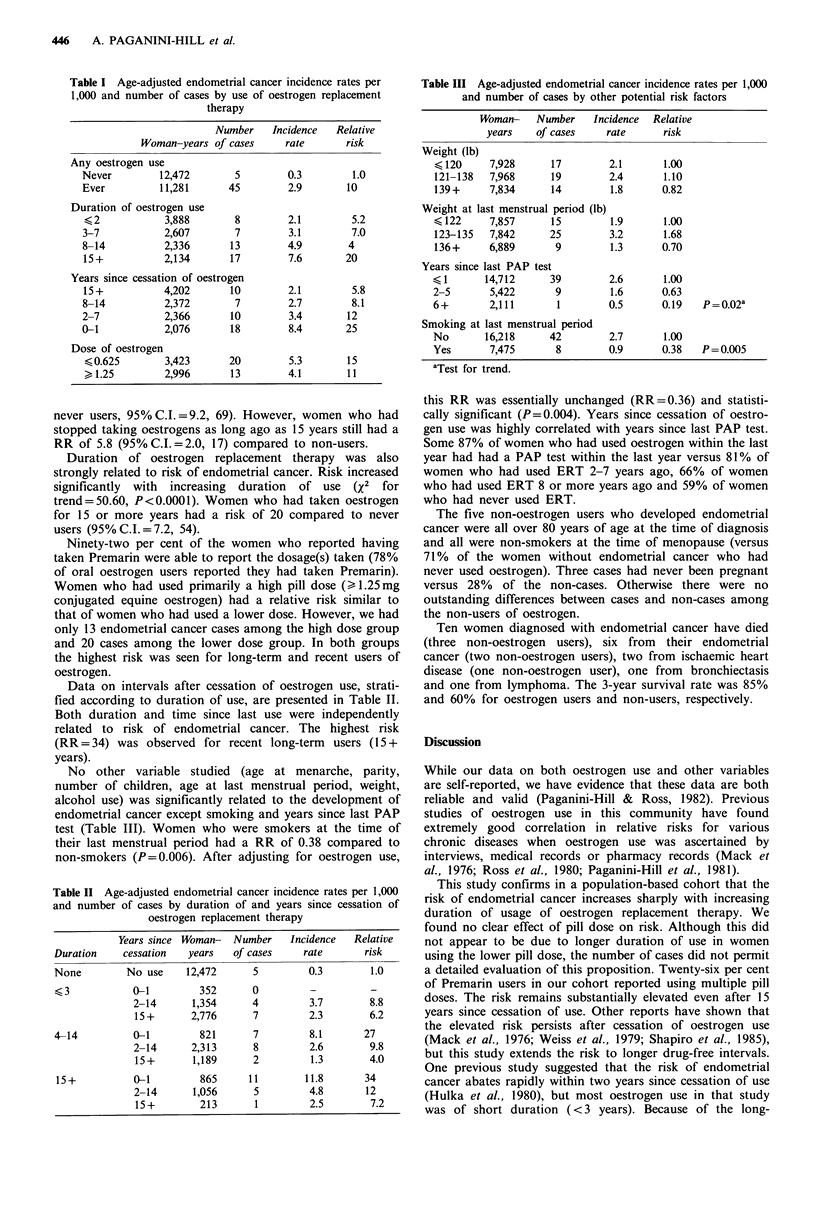

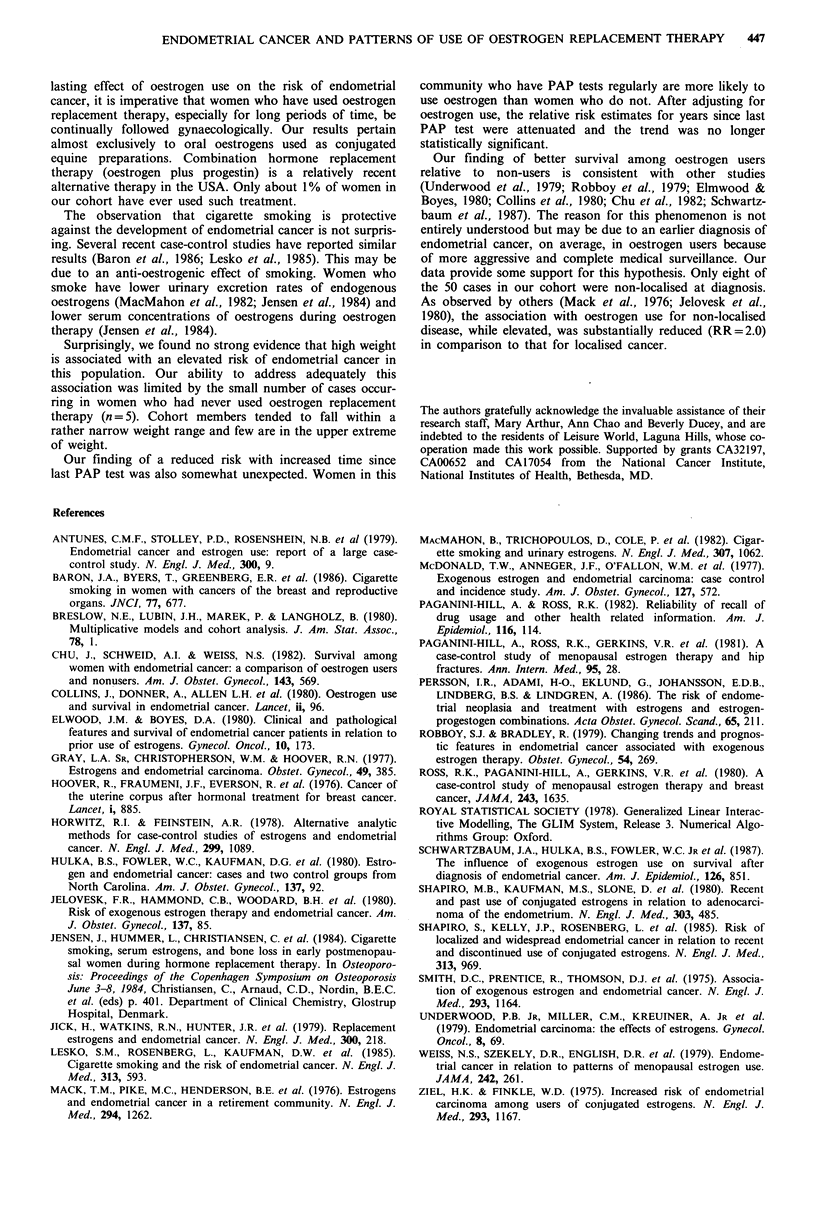


## References

[OCR_00414] Antunes C. M., Strolley P. D., Rosenshein N. B., Davies J. L., Tonascia J. A., Brown C., Burnett L., Rutledge A., Pokempner M., Garcia R. (1979). Endometrial cancer and estrogen use. Report of a large case-control study.. N Engl J Med.

[OCR_00419] Baron J. A., Byers T., Greenberg E. R., Cummings K. M., Swanson M. (1986). Cigarette smoking in women with cancers of the breast and reproductive organs.. J Natl Cancer Inst.

[OCR_00429] Chu J., Schweid A. I., Weiss N. S. (1982). Survival among women with endometrial cancer: a comparison of estrogen users and nonusers.. Am J Obstet Gynecol.

[OCR_00438] Elwood J. M., Boyes D. A. (1980). Clinical and pathological features and survival of endometrial cancer patients in relation to prior use of estrogens.. Gynecol Oncol.

[OCR_00443] Gray L. A., Christopherson W. M., Hoover R. N. (1977). Estrogens and endometrial carcinoma.. Obstet Gynecol.

[OCR_00446] Hoover R., Fraumeni J. F., Everson R., Myers M. H. (1976). Cancer of the uterine corpus after hormonal treatment for breast cancer.. Lancet.

[OCR_00451] Horwitz R. I., Feinstein A. R. (1978). Alternative analytic methods for case-control studies of estrogens and endometrial cancer.. N Engl J Med.

[OCR_00456] Hulka B. S., Fowler W. C., Kaufman D. G., Grimson R. C., Greenberg B. G., Hogue C. J., Berger G. S., Pulliam C. C. (1980). Estrogen and endometrial cancer: cases and two control groups from North Carolina.. Am J Obstet Gynecol.

[OCR_00461] Jelovsek F. R., Hammond C. B., Woodard B. H., Draffin R., Lee K. L., Creasman W. T., Parker R. T. (1980). Risk of exogenous estrogen therapy and endometrial cancer.. Am J Obstet Gynecol.

[OCR_00475] Jick H., Watkins R. N., Hunter J. R., Dinan B. J., Madsen S., Rothman K. J., Walker A. M. (1979). Replacement estrogens and endometrial cancer.. N Engl J Med.

[OCR_00479] Lesko S. M., Rosenberg L., Kaufman D. W., Helmrich S. P., Miller D. R., Strom B., Schottenfeld D., Rosenshein N. B., Knapp R. C., Lewis J. (1985). Cigarette smoking and the risk of endometrial cancer.. N Engl J Med.

[OCR_00489] MacMahon B., Trichopoulos D., Cole P., Brown J. (1982). Cigarette smoking and urinary estrogens.. N Engl J Med.

[OCR_00484] Mack T. M., Pike M. C., Henderson B. E., Pfeffer R. I., Gerkins V. R., Arthur M., Brown S. E. (1976). Estrogens and endometrial cancer in a retirement community.. N Engl J Med.

[OCR_00492] McDonald T. W., Annegers J. F., O'Fallon W. M., Dockerty M. B., Malkasian G. D., Kurland L. T. (1977). Exogenous estrogen and endometrial carcinoma: case-control and incidence study.. Am J Obstet Gynecol.

[OCR_00502] Paganini-Hill A., Ross R. K., Gerkins V. R., Henderson B. E., Arthur M., Mack T. M. (1981). Menopausal estrogen therapy and hip fractures.. Ann Intern Med.

[OCR_00497] Paganini-Hill A., Ross R. K. (1982). Reliability of recall of drug usage and other health-related information.. Am J Epidemiol.

[OCR_00507] Persson I. R., Adami H. O., Eklund G., Johansson E. D., Lindberg B. S., Lindgren A. (1986). The risk of endometrial neoplasia and treatment with estrogens and estrogen-progestogen combinations. First results of a cohort study after one to four completed years of observation.. Acta Obstet Gynecol Scand.

[OCR_00512] Robboy S. J., Bradley R. (1979). Changing trends and prognostic features in endometrial cancer associated with exogenous estrogen therapy.. Obstet Gynecol.

[OCR_00517] Ross R. K., Paganini-Hill A., Gerkins V. R., Mack T. M., Pfeffer R., Arthur M., Henderson B. E. (1980). A case-control study of menopausal estrogen therapy and breast cancer.. JAMA.

[OCR_00527] Schwartzbaum J. A., Hulka B. S., Fowler W. C., Kaufman D. G., Hoberman D. (1987). The influence of exogenous estrogen use on survival after diagnosis of endometrial cancer.. Am J Epidemiol.

[OCR_00532] Shapiro S., Kaufman D. W., Slone D., Rosenberg L., Miettinen O. S., Stolley P. D., Rosenshein N. B., Watring W. G., Leavitt T., Knapp R. C. (1980). Recent and past use of conjugated estrogens in relation to adenocarcinoma of the endometrium.. N Engl J Med.

[OCR_00537] Shapiro S., Kelly J. P., Rosenberg L., Kaufman D. W., Helmrich S. P., Rosenshein N. B., Lewis J. L., Knapp R. C., Stolley P. D., Schottenfeld D. (1985). Risk of localized and widespread endometrial cancer in relation to recent and discontinued use of conjugated estrogens.. N Engl J Med.

[OCR_00543] Smith D. C., Prentice R., Thompson D. J., Herrmann W. L. (1975). Association of exogenous estrogen and endometrial carcinoma.. N Engl J Med.

[OCR_00553] Weiss N. S., Szekely D. R., English D. R., Schweid A. I. (1979). Endometrial cancer in relation to patterns of menopausal estrogen use.. JAMA.

[OCR_00558] Ziel H. K., Finkle W. D. (1975). Increased risk of endometrial carcinoma among users of conjugated estrogens.. N Engl J Med.

